# Flower Mimics Roll Out Multicolored Carpets to Lure and Kill the House Fly

**DOI:** 10.3390/insects12121097

**Published:** 2021-12-08

**Authors:** Hamady Dieng, Tomomitsu Satho, Nor Hafisa Syafina Binti Mohd Radzi, Fatimah Abang, Nur Faeza A. Kassim, Wan Fatma Zuharah, Nur Aida Hashim, Ronald E. Morales Vargas, Noppawan P. Morales

**Affiliations:** 1 Mosquito Research and Control Unit (MRCU), George Town KY1-1106, Cayman Islands; 2 Institute of Biodiversity and Environmental Conservation, Universiti Malaysia Sarawak, Kota Samarahan 94300, Malaysia; 3 Faculty of Pharmaceutical Sciences, Fukuoka University, Nanakuma Jonan-Ku, Fukuoka 814-0180, Japan; satho@fukuoka-u.ac.jp; 4 Faculty of Resource Science and Technology, Universiti Malaysia Sarawak, Kota Samarahan 94300, Malaysia; norhafisasyafina@gmail.com (N.H.S.B.M.R.); fatim@cans.unimas.my (F.A.); 5 School of Biological Sciences, Universiti Sains Malaysia, Gelugor 11800, Malaysia; nurfaeza@usm.my (N.F.A.K.); wfatma@usm.my (W.F.Z.); 6 School of Food Science and Technology, Universiti Malaysia Terengganu, Kuala Terengganu 21300, Malaysia; aida.hashim@umt.edu.my; 7 Faculty of Tropical Medicine, Mahidol University, Krung Thep Maha Nakhon 10400, Thailand; ronald.mor@mahidol.ac.th; 8 Faculty of Science, Mahidol University, Krung Thep Maha Nakhon 10400, Thailand; noppawan.phu@mahidol.ac.th

**Keywords:** housefly, flower arrangement, boric acid, sugar, mortality

## Abstract

**Simple Summary:**

Besides being a nuisance, house flies are known mechanical vectors of bacteria, helminthes, protozoans, and viruses, potentially including Coronaviruses. To prevent the occurrence of these public health issues, efforts have mainly targeted reducing house fly populations with chemical insecticides. However, the development of resistance has impeded success using this strategy. Toxic bait formulations and traps have been crucial components of these control efforts. Although bait-based strategies have sometimes been successful against fly populations, overall, management programs based on this strategy have severely suffered from the development of resistance and lack of attractiveness to the insects. Trapping strategies require the bait to be attuned to the tastes of the target animal. As flies are regular visitors and pollinators that use flowers for numerous other rewards, strategies using blooms as lures may prove effective in managing their populations. Floral mimics have been successfully used to establish preference patterns of insect pollinators. Using dual-choice bioassays with blue-, yellow-, red-, purple-, and pink-colored flowers, we found that colorful designs made of such artificial flowers incorporating a toxicant attracted and killed foraging houseflies. Such attraction of the colorful floral designs suggests the potential for development of sound attract-and-kill devices or strategies integrating artificial flower combinations.

**Abstract:**

Flowers and their spatial clustering are important parameters that mediate the foraging behavior and visitation rate of pollinating insects. Visual stimuli are crucial for triggering behavioral changes in the house fly, *Musca domestica*, which regularly visits plants for feeding and reproduction. The success of bait technology, which is the principal means of combatting flies, is adversely affected by reduced attractiveness and ineffective application techniques. Despite evidence that house flies have color vision capacity, respond to flowers, and exhibit color and pattern preference, the potential of artificial flowers as attractive factors has not been explored. The present study was performed to investigate whether artificial floral designs can lure and kill house flies. Starved wild house flies were presented with equal opportunities to acquire sugar meals, to which boric acid had been added as a toxin, from one flower arrangement (blue-dominated design, BDD; yellow-dominated design, YDD; or pink-dominated design, PDD), and a non-toxic white design (WDD). We also allowed house flies to forage within an enclosure containing two non-toxic floral designs (WDDs). The differences in mortality between the two environments with and without toxicant were examined. The survival rate of *Musca domestica* was extremely high when WDDs containing non-toxic sugar sources were the only feeding sites available. When given an option to forage in an environment containing a BDD and a WDD, house flies showed a high mortality rate (76%) compared to their counterparts maintained in the WDD environment (2%). When kept in an enclosure containing one YDD and a WDD, flies showed a mortality rate of 88%; however, no mortality occurred among flies confined to a compound with a WDD pair. When provided an even chance of foraging in an enclosure containing a mixed pair of floral arrangements (PDD and WDD) and another with two WDDs, flies showed a higher mortality rate (78%) in the first environment. However, the maximum survival rate (100%) was seen in the WDD environment. Exposure to YDD tended to result in a greater mortality rate than with the two other floral designs. Mortality gradually increased with time among flies exposed to tested artificial floral designs. The results presented here clearly indicated that artificial flower arrangements with a toxic sugar reward were strikingly attractive for house flies when their preferred color (white) was present. These observations offer novel possibilities for future development of flower mimic-based house fly control.

## 1. Introduction

House flies (Diptera: Muscidae: *Musca domestica*) thrive in close association with humans [1] and livestock [2], feeding on foodstuff [3,4] where they can pick up and carry a variety of pathogens. In addition to being a nuisance, these flies are mechanical vectors of bacteria, helminthes, protozoans, and viruses to humans [5]. There is accumulating evidence that these flies may be involved as mechanical vectors of Coronaviruses and in the contamination of food crops [6,7,8].

To prevent the occurrence of these public health issues as well as the nuisance factors associated with the house fly, efforts to reduce housefly populations have mostly employed chemical insecticides [9]. However, the development of resistance to most existing chemical classes, e.g., organochlorines, organophosphates, carbamates, and pyrethroids, has impeded the success of such programs [10]. Toxic bait formulations [11] and traps [12] have been crucial components of these control efforts [13]. Although bait-based strategies have sometimes been successful [14] and helped to mitigate adverse health effects associated with fly populations [15,16], overall, management programs based on this strategy have severely suffered from the development of resistance [10] and lack of attractiveness to the insects [9]. Other strategies involving sticky surfaces, bait, and light traps [1,17] or insecticide-treated fly cords [13] were insufficient to manage fly populations [18,19]. This situation has led many groups to suggest that it is necessary to develop novel control strategies [9,10,16].

Some authors have argued for the need to develop novel application strategies [9,15] and maximized attraction factors [16]. Trapping strategies require the bait to be attuned to the tastes of the target animal. In such strategies targeting flies, it makes sense to present them with their key needs. During their life cycle, flies need food, mating opportunities, shelter, and egg laying sites. In addition to visiting flowers for mating and oviposition [20,21,22], house flies interact with plants to obtain sugar from nectar [10]. Plant nectar is a complex mixture, the major components of which are sucrose, glucose, and fructose [23], and is a primary energy source for pollinators [24], which is necessary for sustenance [25], longevity [26], and survival [27]. As flies use flowers for numerous rewards, strategies using blooms as lures may prove effective in housefly management.

There is optimism that flowers may be useful for controlling the house fly, as they are regular visitors and pollinators of over 500 flowering plants [21]. Flies, which are diurnal [28], use visual cues, particularly color, for flower visitation [29] and changes in activity patterns [9]. Their high visual capacity has been attributed to their visual system. Over half of the surface of the house fly head comprises two large compound eyes accompanied by a cluster of three ocelli [18], which confer the ability to respond to different colors [19] with sensitivity ranging from wavelengths of 340 to 540 nm [30,31]. House flies have five absorbance peaks at 335, 355, 460, 490, and 530 [30,31] and exhibit color preferences [14]. A number of studies have shown successful attraction of adult house flies by visual objects in the absence of olfactory ingredients. For example, Waterhouse [32] reported a preference for dark over light colors based on experiments involving presenting houseflies with painted plywood surfaces. Diclaro II et al. [18] demonstrated color preference in house flies by exposing adults to twin-walled, rigid plastic sheets of different colors.

Much of the interest in flowers is based on the results of a study showing that flies respond equally to live flowers and to their mimics [33]. Such floral mimics have also been used to establish ecologically pertinent cognition and preference patterns of insect pollinators [34,35,36,37,38] including mosquitoes [39]. Recently, Khan et al. [9] reported increased attractiveness to house flies of fabrics of different colors treated with insecticide paired with a sugar source. Despite evidence that house flies can be enticed by colored materials, including those with a flower-like configuration [33] or those with toxic nectar mimic [9] the role of artificial flowers with regard to their influence on foraging activity of the house fly has yet to be investigated. The present study was performed to determine whether artificial floral designs with a sweet toxic reward can lure and kill house flies.

## 2. Materials and Methods

### 2.1. Collection of Experimental House Flies

Adults of the Borneo strain of *Musca domestica* were collected from around dustbins and garbage containers in the Malaysian district of Kota Samarahan (1°27′34″ N, 110°29′56″ E), as reported elsewhere [38]. Wild-caught house flies were brought to the insectarium of the Faculty of Resource Science and Technology (Universiti Malaysia Sarawak, Kota Samarahan) and kept under controlled environmental conditions with temperature of 26 °C–28 °C, 75–85% relative humidity, and 14:10 h L:D photoperiod. Adults were kept at densities of approximately 20–50 flies in Bugdorm cages (30 × 30 × 30 cm; MegaView Science Co., Ltd., Taichung, Taiwan) with the continuous presence of sucrose solution (10%). Adults that had been maintained on a sugar diet for 2 days were starved for 12 h and used in the experiments. The same collection and maintenance procedures were repeated to obtain adequate numbers of experimental house flies.

### 2.2. Artificial Flowers and Experimental Flower Arrangements

Floral mimics have been used successfully to establish ecologically relevant behaviors and color preferences of bees and many other pollinating insect groups both in the laboratory [35,39,40,41,42] and in the wild [43]. High-quality artificial tulips (Super Save Co., Ltd., Kuching, Malaysia) were used in this study as floral mimics. Five petal colors documented to be enticing to insect pollinators were selected: three primary colors (yellow, blue, and red) and two secondary colors (pink and purple). Yellow has been reported to be highly attractive to many insects [44,45,46], including house flies [18,47], and has been used to design effective control devices [46,48]. Blue objects have also been reported to be enticing to insects [44,49,50], including houseflies [12,18,32]. Red objects were reported to be attractive to houseflies [9,18]. Purple or pink color flowers have been shown to be highly enticing to key pollinators [51,52].

**Figure 1:**
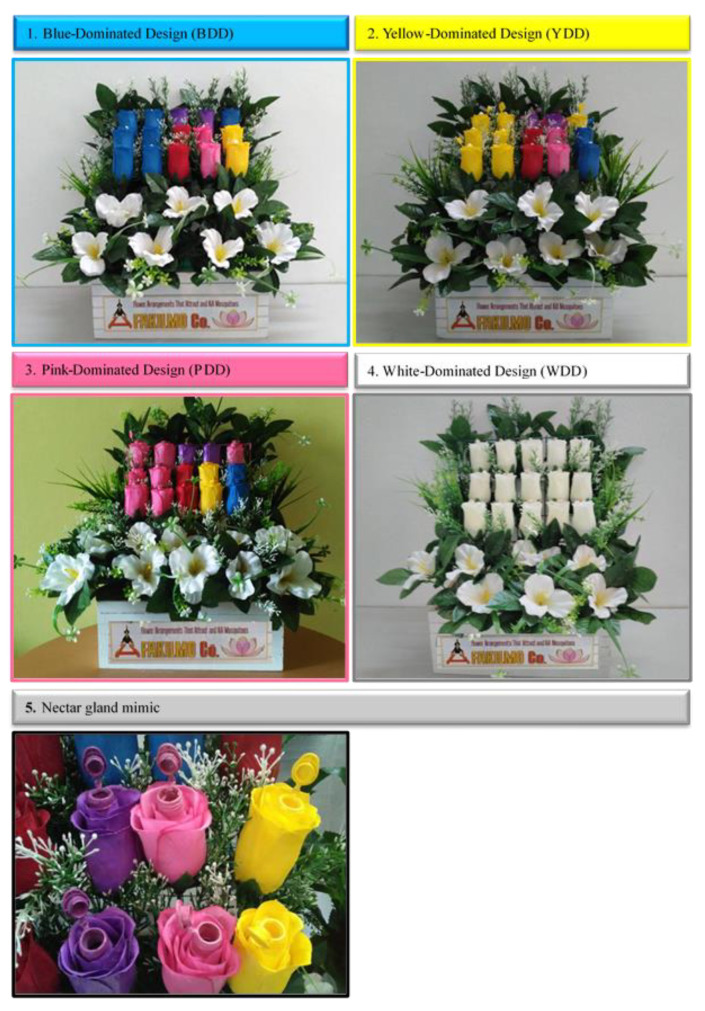
The experimental artificial floral designs used in this study. (**1**). Blue-dominated design (BDD). (**2**). Yellow-dominated design (YDD). (**3**). Pink-dominated design (PDD). (**4**). White-dominated design (WDD). (**5**). Nectar gland mimic present in all individual artificial flowers. BDD, YDD, and PDD were test floral designs, while WDD was the control. All these designs contained 15 artificial flowers arranged in a 3 × 5 design with the same amount of background greenery. All test floral designs possessed the same number of displayed colors but in varying proportions.

The descriptions of artificial tulips and floral arrangements used in the study are presented in Figure 1. Each single flower had a stem, a receptacle, four sepals, and five petals. Each single flower was equipped with a nectar gland mimic consisting of an Eppendorf tube (capacity: 1.5 mL) filled with experimental nectar consisting of aqueous sucrose solution (7.5%) to which boric acid (2.5%) had been added as a toxin; this agent was selected due to its successful use in attractive toxic sugar bait technology [53,54,55,56]. No boric acid was added to white single flowers. The tubes were spray-painted using Samurai Aerosol spray paint (SAMURAI^®^, Johor, Malaysia). A cotton wick stick (4 cm in length) was placed at the bottom inside the tube to act as a nectar gland mimic and floral reward source, as reported elsewhere [57,58]. A 10% sugar solution was prepared to serve as a control [59]. The nectar gland mimic was placed inside the flower at the point of connection of the petals.

### 2.3. Production of Floral Designs

**Figure 2:**
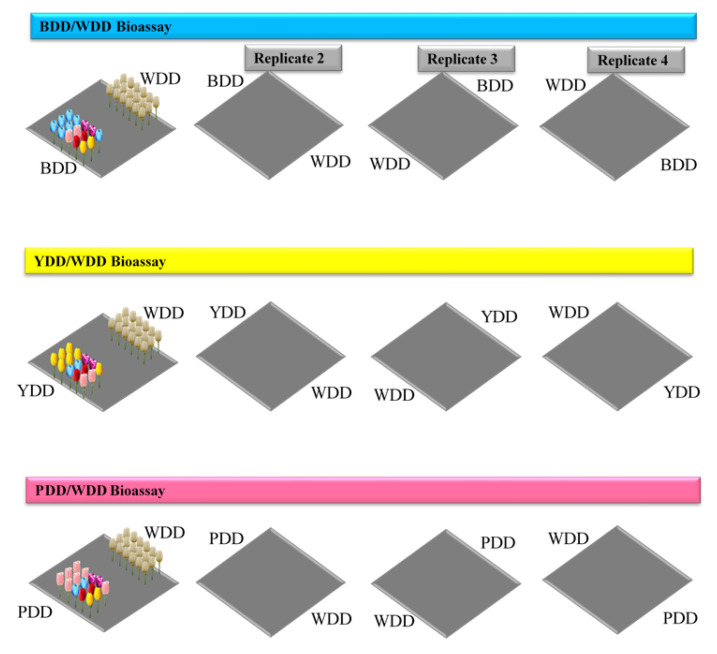
Bioassay layout. The two flower arrangements to be tested were placed at two opposite sides of the enclosure. To prevent position bias, we ran a dual-choice test design following a clockwise replication system, in which a test replicate corresponded to the disposition of two floral designs (one test design and one control design). The same replication strategy was also applied to control enclosures (two WDDs).

Figure 2 shows the configurations of the experimental floral arrangements. Three test floral designs were made using the selected floral mimics: blue-dominated design (BDD), yellow-dominated design (YDD), and pink-dominated design (PDD). White petals structurally similar to the five chosen colored petals were used to obtain a flower arrangement with a white-dominated design (WDD). We used “WDD” as a control based on the increased attractiveness of the white color to flies [60], including houseflies [18,32]. All test floral designs contained 15 flowers laid out in a 3 × 5 pattern with the same amount of background greenery. They all displayed the same number of colors but in different proportions: BDD had seven blue flowers and two flowers for each of the four other colors (yellow, purple, pink, and red); YDD had seven yellow flowers and two each of the other four colors; PDD had seven pink flowers and two each of the other four colors; in WDD (control), all 15 flowers were white.

### 2.4. Bioassays

To determine the attractiveness and lethality of artificial floral layouts, adult house flies were given equal choices between colorful flowers with a toxicant and WDDs. Experimental groups of 15 to 28 wild-caught flies that had been fed sugar for 2 days and starved for half a day were released into an enclosure (1 × 1 × 1 m) containing a BDD at one side and a WDD at the opposite side. Similarly, the same numbers of flies were released into another enclosure containing two WDDs (control cage). On other days, three additional replicates of each of the two treatments (1 BDD + 1 WDD + flies, and 1 WDD + 1 WDD + flies) were set up as outlined above. At other times, the same experimental design, number of flies, and enclosure replicates reported for “BDD/WDD” were also applied for the two other test floral arrangements; the two groups of flies are represented as “1 YDD + 1 WDD” and “1 PDD + 1 WDD” in the YDD and PDD bioassays, respectively. Each of these bioassays was associated with a control enclosure (1 WDD + 1 WDD + flies) according to the same test design and procedures as for the control enclosure for the three bioassays (one bioassay for each of the three test floral designs) (Table 1). To avert position bias, we adopted a dual-choice test design following a clockwise replication strategy, as reported elsewhere [61] with slight modifications. A given replicate coincided with the disposition of two floral designs (one test multicolored design and one control design). The same replication strategy was also applied to control enclosures (2 WDDs). All observations were conducted during the day (12:00 to 16:00) in the laboratory (26 °C–28 °C, 75–85% relative humidity, and 14:10 h L:D photoperiod).

### 2.5. Data Collection and Statistical Analysis

After setting up a bioassay, the total numbers of dead flies were counted for each enclosure replicate (test: colorful floral design/WDD; control: WDD/WDD) at different time points (24, 48, and 72 h). Any fly that was immobile and dry to the touch was recorded as dead. These counts were used to determine fly mortality rates, calculated as the number of dead flies divided by the initial number of flies exposed to a given test floral design) × 100. This calculation was performed for each enclosure replicate and time point, and the resulting mean values were scored as mortality rates. The differences in mortality responses were detected by non-parametric (Kruskal–Wallis) and parametric (analysis of variance) tests using the Systat v.11 statistical software package [62]. Tukey’s post hoc and Dwass–Steel–Chritchlow–Fligner tests were used to compare the differences between exposure times. In all analyses, *p* < 0.05 was taken to indicate statistical significance. 

## 3. Results

### 3.1. BDD and House Fly Mortality Rates

**Figure 3:**
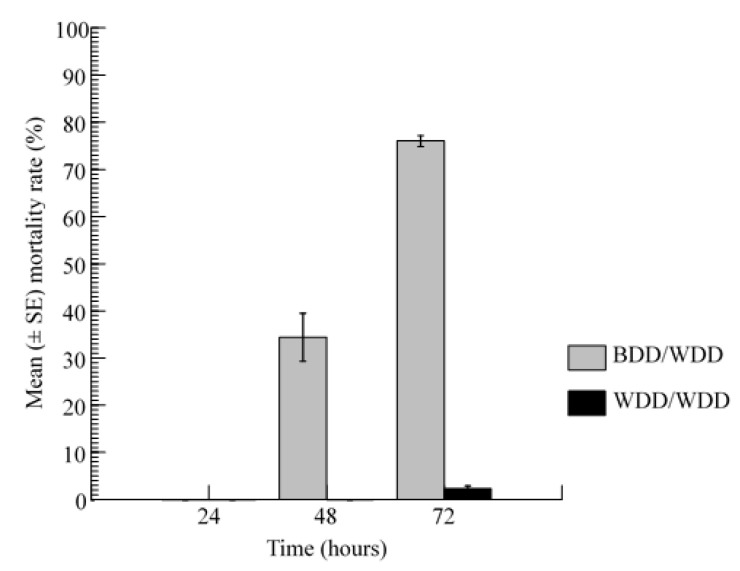
Mortality rates of the adult house flies when allowed to forage in an enclosure with a mixed pair of floral arrangements (one BDD delivering toxic sugar and one WDD with non-toxic sugar) and another enclosure with a pair of WDDs.

House flies maintained in the enclosure with two WDDs (bearing non-toxic sugar sources) survived at high rates throughout the 3-day trial period (97.2 ± 2.33%; range: 86–100%); only one house fly was found dead after 72 h. In the BDD/WDD enclosure, the mortality responses of the house fly varied significantly with time (Kruskal–Wallis test statistic = 7.227, df = 2, *p* = 0.027), with the 72 h exposure producing the highest rate (76.02 ± 4.65; range: 65–87.5). No fly died after 24 h, and the mean mortality rate of flies after 48 h (34.42 ± 20.2; range: 0–77.7) was 2.20 times lower than that recorded after 72 h, which, in turn, was far greater than that after 24 h (Dwass–Steel–Chritchlow–Fligner Test Pairwise Comparisons (DSCF) = 3.480, *p* = 0.037) (Figure 3).

### 3.2. YDD and House Fly Mortality Patterns

**Figure 4:**
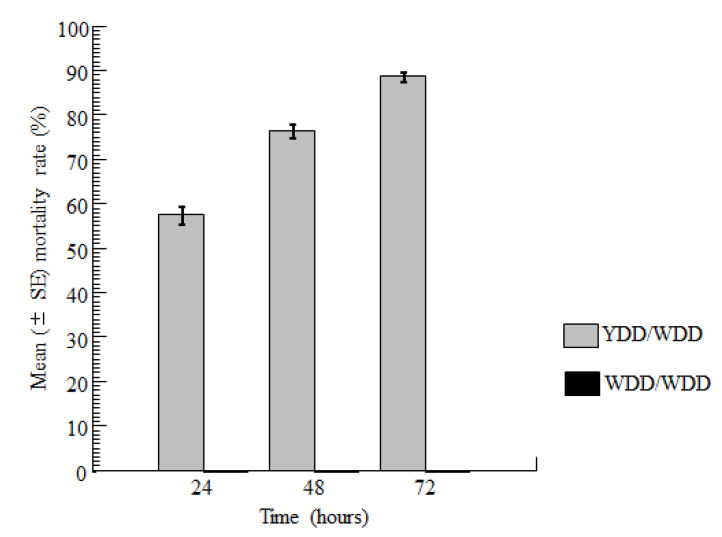
Mortality rates of the house flies when provided feeding opportunities in an enclosure with an assorted pair of floral arrangements (one YDD bearing toxic sugar and one WDD with non-toxic sugar) and another enclosure with paired WDDs.

All house flies that were kept in an enclosure containing two WDDs survived throughout the observation period. However, in the YDD enclosure (holding one YDD in competition with one WDD), there were significant temporal differences in fly mortality response (F = 5.95, df = 2, *p* = 0.022). In this enclosure, the mortality rate after 24 h (57.5 ± 8.03%; range: 40.9–78.26%) was 1.32 times lower compared to that obtained after 48 h (76.42 ± 6.39%; range: 63.6–91.3%), which, in turn, was 1.16 times lower than that recorded on day 3 (88.73 ± 4.37%; range: 81.8–100%). There were no significant differences in mortality rate between 24 and 48 h-exposures (Matrix of pairwise mean differences (MPMD) = −18.922, *p* = 0.150). In contrast, house flies died at an appreciably higher rate on day 3 compared to day 2 (MPMD = −31.225, *p* = 0.019) (Figure 4).

### 3.3. PDD and House Fly Mortality Rates

**Figure 5:**
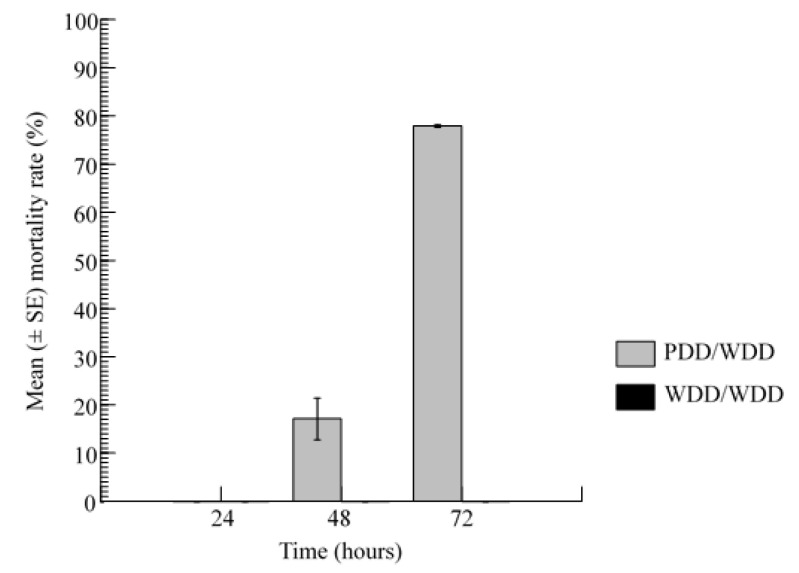
Mortality rates of the house flies when they were placed in two different environments: one enclosure containing an assorted pair of floral designs (one PDD with toxic sugar and a WDD with non-toxic sugar) and a control enclosure containing two WDDs.

When adult house flies were confined in an enclosure containing a PDD and a WDD, and another with two WDDs (control), the mortality response showed significant temporal variations (Kruskal–Wallis Test Statistic = 9.374, df = 2, *p* = 0.009), with no dead flies in the control enclosure throughout the the 3-day observation period. In the test enclosures, the mortality response after 24 h tended to be lower compared to day 2 (13 dead out of 80), but the difference between the two first days of exposure was insignificant (DSCF = 1.414, *p* = 0.577). The mean mortality response of the house fly after 72 h of exposure (77.91 ± 1.07%; range: 75–80%) was significantly higher than those obtained 24 h (*DSCF* = 3.480, *p* = 0.037) and 48 h (DSCF = 3.347, *p* = 0.047) after the commencement of the trial (Figure 5).

## 4. Discussion

The results of the present study indicated that house flies feed on toxic nectar mimics held by artificial floral designs. Using dual-choice bioassays with blue-, yellow-, red-, purple-, and pink-colored flowers, our observations indicated that colorful designs made of such artificial flowers incorporating a toxicant (i.e., BDD, YDD, and PDD) attracted and killed foraging house flies. This was the first formal study to document the behavioral significance of artificial colored flowers and the lethality of their assemblages for *M. domestica*.

Before discussing the findings in more detail, we will first address our methodology to provide the necessary background. All flower mimics used in this study were of colors reported to be enticing to insect pollinators either under field or controlled laboratory conditions. As our ultimate goal is to improve bait technology with flowers as lures, we used floral designs with diverse colored flowers. In nature, flower density has often been shown to be associated with the rates of pollinator visitation, and floral color changes are believed to occur to facilitate pollination by diversifying floral displays of monospecific stands [63,64]. In bees, Ye et al. [65] observed high visitation rates in plots with increased floral densities. Clearly, a diversity of flowers will result in increased cumulative attraction and benefits for pollinators. With a diversity of flowers present within a community, differential attractions of each of the flowers will combine to produce a collective floral attraction. As reported previously [64,66], the presence of many flowers represents greater amounts of floral resources for pollinating insects. Ghazhoul [64] reported that facilitation of pollination mostly occurs among plant species that have similar floral forms. In the present study, all single flowers used to make the floral designs were similar in structure; each flower possessed a stem, a receptacle, four sepals, and five petals made of polyester fabric and paper [37,67]. Flowers made of similar materials have been shown to be visited by flies at rates similar to those of real flowers [33]. Based on the abovementioned reports and the structural uniformity of the experimental flowers, we believe that our findings were not artefacts.

About 76% of adult house flies died when allowed to forage within an enclosure containing a BDD with a toxic sugar reward and a WDD with a non-toxic sugar source for 3 days. However, >97% of their counterparts in the control enclosure containing two WDDs were still alive after the same exposure period. Previous studies have shown that white is highly attractive to flies [68]. Pickering and Stock [60] tested five colored traps and reported increased preference of house flies for white over yellow, orange, red, and purple. In a similar study, Waterhouse [32] examined the impacts of many plywood surfaces of various colors on the resting preference of the house fly and noted considerably higher resting rates on white surfaces over blue, gray, green, yellow, and red surfaces. Diclaro II et al. [18] examined the behavioral responses of house flies to colored targets and reported that white was more effective as a visual attractant than yellow or blue. Blue fabric targets have been shown to be more enticing to house flies than white and black targets [12].

In the BDD used in the present study, 46.6% (7/15) were blue flowers, with two each of yellow, purple, pink, and red flowers. Blue color has been reported to be highly attractive to insect pollinators. For example, Raine and Chittka [69] inspected color preference of the bumblebee *Bombus terrestris* from nine colonies by presenting them with artificial flowers of different colors and nectar contents. They found increased preference for blue flowers, which they ascribed to nectar rewards and sugar contents. Recently, Acharya et al. [70] examined four different colors of pan traps for their utility in sampling bees, and found significant more captured subjects in blue traps than green, yellow and purple traps. Moyroud et al. [71] reported that hundreds of flower species have evolved the ability to produce nanostructures that produce a blue halo to lure pollinating bees. Flies have compound eyes with spectral sensitivity of 310–700 nm [72] and visual pigments that respond maximally to blue-green with an absorbance peak at 490 nm [73,74]. The different research outcomes taken together and recent records demonstrating elevated attraction of flies to floral mimics [33] and fabrics paired with sugar suspension [9] and the preference for blue color [12,18] altogether seem to suggest that the house fly was attracted to BDD, as it related blue with elevated sugar reward. It is also likely that the appreciable level of visitation and subsequent mortality observed with BDD/WDD incorporated the differential attractions of each of the other colored flowers within BDD, as complementary attraction usually occurs when flowers within mixed floral displays are similar [64].

The mortality rate was relatively high (88%) when flies were given an opportunity to feed on sugar in the enclosure containing YDD and WDD; in contrast, maximal survival was seen in the enclosure containing a pair of colorless floral displays (WDDs). The majority of flowers within YDD were yellow in color (46.6%), with the remainder composed of two of each of the other colors (red, purple, pink, and blue). It is commonly though that many insect pests are enticed to yellow plants, because this color advertises stress, weakened defenses, and thus greater feeding opportunities. Many previous studies confirmed the attractiveness of color yellow to houseflies using test materials composed of paper [75], plastic [47], or card [76,77]. Yellow sticky traps have been used with great success in the control of many insects [46,78,79,80], including the house fly [12]. This technology is based on the natural attractiveness of the yellow color to insects, most of which are day-active [48]. As in many other diurnal species, the housefly has an absorbance peak at 570 nm, which corresponds to yellow [73]. Sensitivity to yellow wavelengths has been confirmed electrophysiologically [18]. These latter authors reported that flies were attracted far more strongly to blue than to yellow, which tended to repel them. They also reported that yellow targets were appreciably less enticing and almost repellent to house flies when in competition with white or blue targets. In a related study, Waterhouse [32] also reported a similar unattractiveness of yellow surfaces, which was attributed to the brightness of the yellow color to the flies. Based on the above-mentioned reports, it is possible that the observed increased mortality rate observed in the YDD/WDD enclosure occurred due to the collective attraction in which the combination of the singular attractions of the seven yellow flowers within YDD resulted in a cumulative strong attraction to flies, which subsequently fed on toxic nectar and died. It is also likely that the presence of the other eight flowers contributed to YDD attraction to the house flies.

House flies enclosed with a PDD and a WDD died at a high rate (77.9%), whereas no deaths were recorded in the enclosure containing two WDDs, indicating that the presence of PDD did have a negative impact on survival of house flies. Discrepancies in sugar feeding intensity have usually been linked to nectar sugar availability, which, in turn, has been associated with flower color in insect pollinators [49,69,81,82]. Culin [51] examined the effects of several flower cultivars with different colors and nectar reward potentials on the visitation intensity of a butterfly and observed an increased preference for pink flowers over pale or white flowers. They attributed this attractiveness to high quantities of nectar and sugar sources. Similar to butterflies, house flies are also pollinators [21,29]. These two insect groups have compound eyes, but they differ in size and sensitivity. Butterflies have a spectral sensitivity of 370–570 nm [83], whereas house flies are able to see wavelengths from 310 to 700 nm [72]. There is evidence that flies can identify floral cues [9,84], including floral shape [33], and link optical signals with the presence or absence of sugar [9]. In the present study, we used a pink floral display (PDD) and a fully white competitor (WDD), with an enclosure containing a pair of WDDs as a control. White color has often been documented as being highly attractive to nectar-feeding insects [12,18,68]. With reference to these reports, it seems likely that the house fly studied here associates floral colorfulness with sugar reward. These previous reports and our observations suggest that this fly is capable of associating flower color with reward quality, as pinkish flowers indicate increased sugar availability [51,82]. The collective attractions of both the pink flowers and other less highly represented flower colors (two each of red, purple, yellow, and blue) may have played a role in this increased feeding and ensuing mortality rate.

It is interesting to note that the house fly mortality rate exhibited a temporal pattern in all treatments. The mortality rates after 24 h tended to be lower than those at 48 h, which, in turn, were appreciably lower than those recorded after 72 h. Insect pollinators prefer sites where floral resources are readily and highly available [64]. House flies visit flowers for feeding [85], sheltering, and mating [21,22]. In the present study, such differential responses (visitation and subsequent mortality) were unlikely, as both colorful floral arrangements (BDD, YDD, and PDD) and the colorless design (WDD) in the test (colorful vs. WDD) and control (WDD/WDD) enclosures were identical in nectar mimic content. Clearly, white floral displays are less dark than colorful floral displays, which may represent more safe sheltering/hiding sites. Such darker environments are likely to have an impact on mating success. In house flies, mating generally occurs during resting and is initiated by the male, which may be avoided by the female as harassment [86]. However, such mating attempts are likely to be successful in a dark environment. Although we did not sex the experimental flies in the present study, the observed increased morality rates in the enclosure containing colorful floral displays paired with a WDD may have been due to greater visitation and presence on the multicolored floral designs over time.

To our knowledge, this is the first study to evaluate the effects of rewarding artificial flowers of different colors and their collective floral displays on house fly foraging responses. The experiments involved three floral designs with different color configurations and boric acid as a toxicant to examine their potential use in fly bait-based strategies, which are known to suffer from unattractiveness of the bait. Recently, Tiusanen et al. [33] reported increased attractiveness of floral mimics to flies, which was preceded by the discovery that house flies are highly attracted to sugar-rewarding colored fabrics paired with a toxicant. For flies, the major reason for flower visitation is the food reward obtained in the form of sugar primarily from floral nectar [10,22,87]. Pollen protein is essential for reproduction in at least some fly species [88]. However, for house flies, there are also other benefits, including shelter [22] and oviposition sites [20,21]. Flowers can also provide species-specific rendezvous sites for mating [89]. Hence, foraging for such crucially important floral resources has become a valuable target for effective fly control. Many colorful objects, in some cases mimicking the natural resources of the house fly, have been tested or used in strategies to manage its populations. For example, Chow and Thevasagayam [90] designed a portable frame tied with string soaked in insecticide to trick house flies into resting upon it. Khan et al. [9] proposed a combination of a sweet phagostimulant, colorful fabrics, and insecticides as potential toxic bait. Diclaro II et al. [18] designed the fly-Baiter using blue color, sweetness, sexually enticing compounds, and odorants. Baker et al. [16] tested the combination of a sugar and an entomopathogenic fungus to design toxic baits. Other strategies, i.e., sticky and ULV light traps that use color as attractant, have also been developed [1,17]. However, most of these methods did not make use of the diversity of non-nutritive rewards, which may limit their effectiveness. For designs that make use of olfaction, attraction may be hindered by the transient nature of odors [91,92] and the overwhelming amounts of odorous substances in the environment [93,94]. For house flies, which are diurnal [28], light traps may be unattractive. Devices that target only the resting behavior may not attract house flies seeking mates. The floral displays tested in the present study were undeniably enticing to the house fly. Such attraction of the colorful floral designs suggests the potential for development of sound attract-and-kill devices or strategies integrating artificial flower combinations. The development of such tools incorporating boric acid as a toxicant would be practical not only for house flies, but for any insect vector that acquires sugar, mates, or hiding opportunities primarily from flowers. Such artificial flower-based toxic bait using color as an attractant has the potential for long-term persistence, as color is unaffected by wind [95], and many artificial flowers are resistant to fading. Moreover, as flower arrangements are often displayed in human dwelling areas, environments where house flies thrive, the development of floral design-based bait may be a good option.

There are limitations that need to be expressed with respect to our methodology. We assessed the lethality of the different floral designs against the house fly, but we did not investigate visitation patterns. However, as only test colorful floral designs had the toxicant (boric acid), every dead fly recorded in each of the test enclosures had been attracted, surely landed on a floral design, foraged, and fed on the toxic sugar solution. This clearly demonstrated that the test floral designs exhibited an attraction, and that the mortality rates obtained in each test enclosure are in fact visitation rates. There is another factor related to our methodology that should be discussed. Our design consisted of three test enclosures (i.e., BDD vs. WDD, YDD vs. WDD, and PDD vs. WDD) that contained the toxicant (boric acid), and control enclosures (WDD vs. WDD). We assumed that natural mortality record was required to validate the performance of the test floral designs and know what impacts we can reasonably expect to observe if operationally used [96] (Arnold and Ercumen 2016). The data obtained by using two WDDs as control did illustrate that the floral displays overall can entice and kill houseflies. In contrast to negative control, a positive control is a test in which a positive result is expected; to this end, it uses a treatment that is already known to produce that effect (Moser 2019). We had a negative control but not a positive one; however, it is well-know that boric acid is lethal to the house fly, *Musca domestica*, and this effect has been used as a framework to develop sugar baits [59,97].

## Figures and Tables

**Table 1:** Experimental setups.

Study	Enclosure	Replication	Data Collection Time
BDD and house fly mortality responses	Test enclosure: BDD vs. WDD Control enclosure: WDD vs. WDD	4 6	24, 48, 72 hours
YDD and house fly mortality responses	Test enclosure: YDD vs. WDD Control enclosure: WDD vs. WDD	4 5	24, 48, 72 hours
PDD and house fly mortality responses	Test enclosure: PDD vs. WDD Control enclosure: WDD vs. WDD	4 5	24, 48, 72 hours

## Data Availability

The data presented in this study are available on request from the corresponding author.
